# *N*-(2-{[7-(2-Anilinoeth­oxy)-3,6-dibromo­naphthalen-2-yl]­oxy}eth­yl)aniline

**DOI:** 10.1107/S1600536812014808

**Published:** 2012-04-13

**Authors:** Qian-Shou Zong, Jian-Yi Wu

**Affiliations:** a College of Biology and Chemical Engineering, Jiaxing University, Jiaxing, Zhejiang 314001, People’s Republic of China; b School of Pharmaceutical Sciences, Zhejiang University, Hangzhou, Zhejiang 310058, People’s Republic of China

## Abstract

In the title compound, C_26_H_24_Br_2_N_2_O_2_, the central naphthalene system carries two Br atoms and two –CH_2_CH_2_NHC_6_H_5_ substituents. The phenyl rings of the latter residues are inclined at 74.17 (17) and 51.4 (2)° with respect to the naphthalene ring system. Each alkyl chain adopts a fully extended all-*cis* conformation with respect to the naphthalene and phenyl rings [N—C—C—O torsion angles = 68.6 (4) and 60.5 (4)°]. In the crystal, one of the N—H groups forms bifurcated N—H⋯(Br,O) hydrogen bonds, which link the mol­ecules into inversion-related dimers. The centrosymmetric dimers are aggregated *via* pairs of C—H⋯π inter­actions into sheets parallel to (110).

## Related literature

For background information on applications of open-chain crown ethers in extraction and analysis, see: Zhang *et al.* (2002 ▶); Qin *et al.* (2003 ▶); Tan *et al.* (1986 ▶); Chandan, Ved & Kumar (2008 ▶). For related structures, see: Chandan, Ved, Niraj *et al.* (2008 ▶); Liou *et al.* (2011 ▶).
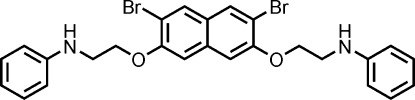


## Experimental

### 

#### Crystal data

C_26_H_24_Br_2_N_2_O_2_
*M* *_r_* = 556.29
Triclinic, 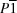

*a* = 9.588 (3) Å
*b* = 10.898 (3) Å
*c* = 13.060 (4) Å
α = 103.190 (4)°
β = 93.953 (4)°
γ = 115.622 (5)°
*V* = 1176.0 (6) Å^3^
*Z* = 2
Mo *K*α radiation
μ = 3.47 mm^−1^
*T* = 296 K
0.30 × 0.20 × 0.12 mm

#### Data collection

Bruker SMART CCD area-detector diffractometer
Absorption correction: multi-scan (*SADABS*; Sheldrick, 1996 ▶) *T*_min_ = 0.439, *T*_max_ = 0.659
9333 measured reflections
4156 independent reflections
2712 reflections with *I* > 2σ(*I*)
*R*_int_ = 0.045

#### Refinement

*R*[*F* ^2^ > 2σ(*F* ^2^)] = 0.042
*wR*(*F* ^2^) = 0.088
*S* = 1.08
4156 reflections
289 parameters
H-atom parameters constrained
Δρ_max_ = 0.48 e Å^−3^
Δρ_min_ = −0.60 e Å^−3^

### 

Data collection: *SMART* (Bruker, 2007 ▶); cell refinement: *SAINT* (Bruker, 2007 ▶); data reduction: *SAINT*; program(s) used to solve structure: *SHELXS97* (Sheldrick, 2008 ▶); program(s) used to refine structure: *SHELXL97* (Sheldrick, 2008 ▶); molecular graphics: *SHELXTL* (Sheldrick, 2008 ▶); software used to prepare material for publication: *SHELXTL*.

## Supplementary Material

Crystal structure: contains datablock(s) I, global. DOI: 10.1107/S1600536812014808/tk5077sup1.cif

Structure factors: contains datablock(s) I. DOI: 10.1107/S1600536812014808/tk5077Isup2.hkl

Supplementary material file. DOI: 10.1107/S1600536812014808/tk5077Isup3.cml

Additional supplementary materials:  crystallographic information; 3D view; checkCIF report

## Figures and Tables

**Table 1:** Hydrogen-bond geometry (Å, °) *Cg*1 and *Cg*2 are the centroids of the C1–C6 and C9–C14 benzene rings, respectively.

*D*—H⋯*A*	*D*—H	H⋯*A*	*D*⋯*A*	*D*—H⋯*A*
N1—H27⋯Br1^i^	0.86	3.03	3.705 (4)	137
N1—H27⋯O2^i^	0.86	2.53	3.349 (4)	160
C5—H5⋯*Cg*2^ii^	0.93	2.81	3.626 (5)	147
C15—H15⋯*Cg*1^iii^	0.93	2.86	3.782 (4)	175

Symmetry codes: (i) 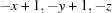; (ii) 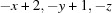; (iii) 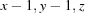.

**Fig. 1.**
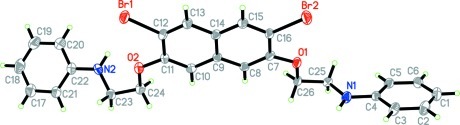
The molecular structure of the title compound, with displacement ellipsoids at the 30% probability level.

**Fig. 2.**
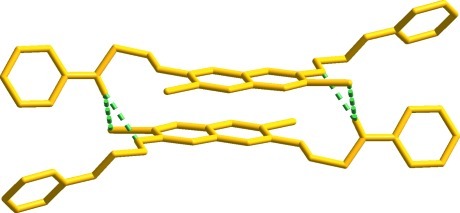
The cyclic hydrogen-bonded dimer in the title compound with hydrogen bonds shown as dashed lines. H atoms, except for those involved in hydrogen bonds, are not included.

**Fig. 3.**
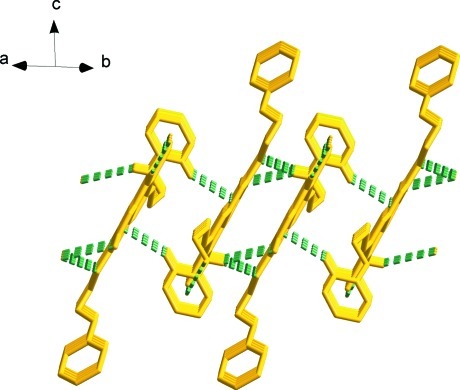
Side-on view of the supramolecular layer parallel to (1 1 0) and mediated by N—H···O and N—H···Br hydrogen bonds and C—H···π interactions plane. H atoms have been omitted for clarity, except for those involved in intermolecular interactions (green dashed lines).

## supplementary crystallographic information

### Comment

Open-chain crown ethers are flexible ligands which offer many advantages in extraction and analysis (ion-selective electrodes) of the rare earth ions (Zhang *et al.*, 2002; Qin *et al.*, 2003; Chandan, Ved, Kumar, 2008; Liou *et al.*, 2011). With suitable substitution at the terminal groups, these molecules can recognize anions or cations and may can also form stable complexes with them (Chandan, Ved, Niraj *et al.*, 2008). In particular, the open-chain crown ethers containing amide groups possess suitable molecular structure for this chemistry: a chain with inflexible terminal groups. Therefore, they are excellent reagents for activating ion-selective electrodes and for extraction of rare earth ions (Tan *et al.*, 1986). In the present work, we designed and synthesized a new and doubly functionalised open-chain crown ether ligand with terminal phenylamine groups, and its crystal structure was determined by X-ray diffraction methods.

As shown in Fig. 1, the naphthalene ring of the molecule contains two —CH_2_CH_2_NHC_6_H_5_ residues and two bromo atoms. The N2-containing —CH_2_CH_2_NHC_6_H_5_ residue is nearly planar with a maximum deviation of 0.065 (9) Å [for atom C23] from the mean plane of its constituent atoms. However, the other residue [having the N1 atom] is less planar with a maximum deviation of 0.18 (6) Å for the C26 atom. The naphthalene ring system (C7-C16) is oriented with respect to the phenyl rings of the N1- and N2- residues at 74.17 (17) and 51.4 (2)°, respectively; the dihedral angle between the terminal phenyl rings is 56.4 (2)°. Both alkyl chains adopt the same fully extended all-*cis* conformation with respect to the naphthalene and phenyl rings [torsion angle: C7–O1–C26–C25 = 172.5 (3), O1–C26–C25–N1 = 68.6 (4), C4–N1–C25–C26 = -165.2 (3), C24–C23–N2–C22 = 179.3 (3), N2–C23–C24–O2 = 60.5 (4), C11–O2–C24–C23 = -177.4 (3)°]. In the crystal, only one of the two N—H groups forms hydrogen bonds. The N1—H27 atom is in fact bifurcated forming N—H···Br and N—H···O hydrogen bonds. These link the molecules into centrosymmetric dimers (Fig. 2 and Table 1). These centrosymmetric dimers are further aggregated *via* pairs of C—H···π interactions into two-dimensional sheets parallel to (1 1 0) (Fig. 3).

### Experimental

To a 50 mL two-necked round-bottom flask equipped with a magnetic stirring bar, a thermometer and a dropping funnel was added 3,6-dibromo-2,7-di((*N*-phenylacetamide)oxy)naphthalene (1.20 g, 2 mmol), sodium borohydride (0.8 g, 22 mmol) and anhydrous tetrahydrofuran (20 mL). The mixture was cooled to 273 K and boron trifluoride etherate (48 %, 4 mL) was added drop-wise via a dropping funnel, while maintaining the temperature at 273 K. After complete addition, the reaction mixture was stirred for one additional hour at 273 K to allow completion of the reaction and then was quenched by drop-wise addition of aqueous NaOH (1 M, 10 mL) while keeping the temperature below 278 K. The tetrahydrofuran was removed under under reduced pressure and the remains were extracted with CH_2_Cl_2_ (3×15 mL). The organic layers were combined and washed with water (10 mL) and then dried over anhydrous Na_2_SO_4_. The solvent was evaporated under reduced pressure to give the title compound as a white solid (1.10 g, 98% yield). Single crystals suitable for X-ray diffraction were obtained by recrystallizing the crude product from its chloroform solution and slow evaporation at room temperature over a period of 5 days; *M*.pt: 378.8–379.5 K. ^1^H NMR (*d*^6^-DMSO, 400 MHz) δ (p.p.m.): 3.63-3.66 (t, 4H), 4.26-4.29 (t, 4H), 6.71-6.77 (q, 6H), 6.979 (s, 2H), 7.23-7.25 (t, 4H), 7.87 (s, 2H). ^13^C NMR (*d*^6^-DMSO, 400 MHz) δ (p.p.m.): 43.13, 67.65, 107.06, 112.02, 113.44, 118.13, 125.63, 129.36, 131.03, 133.65, 147.68, 153.30. MS (ESI) *m/z*: 555.03 [M+H]^+^, 577.01 [M+Na]^+^; Anal. Calcd. (%) for C_26_H_24_Br_2_N_2_O_2_: C, 56.14; H, 4.35; N, 5.04. Found (%): C, 55.12; H, 4.37; N, 5.05.

### Refinement

All H atoms were placed in idealized positions (C—H = 0.93–0.97 Å and N—H = 0.86 Å) and refined as riding atoms with *U*_iso_(H) = 1.2*U*_eq_(C or N).

### Crystal data

**Table:**

C_26_H_24_Br_2_N_2_O_2_	*Z* = 2
*M**_r_* = 556.29	*F*(000) = 560
Triclinic, *P*1	*D*_x_ = 1.571 Mg m^−^^3^
Hall symbol: -P 1	Mo *K*α radiation, λ = 0.71075 Å
*a* = 9.588 (3) Å	Cell parameters from 10579 reflections
*b* = 10.898 (3) Å	θ = 1–28°
*c* = 13.060 (4) Å	µ = 3.47 mm^−^^1^
α = 103.190 (4)°	*T* = 296 K
β = 93.953 (4)°	Cubic, colourless
γ = 115.622 (5)°	0.30 × 0.20 × 0.12 mm
*V* = 1176.0 (6) Å^3^	

### Data collection

**Table:**

Bruker SMART CCD area-detector diffractometer	4156 independent reflections
Radiation source: fine-focus sealed tube	2712 reflections with *I* > 2σ(*I*)
Graphite monochromator	*R*_int_ = 0.045
φ and ω scans	θ_max_ = 25.1°, θ_min_ = 3.1°
Absorption correction: multi-scan (*SADABS*; Sheldrick, 1996)	*h* = −10→11
*T*_min_ = 0.439, *T*_max_ = 0.659	*k* = −11→13
9333 measured reflections	*l* = −15→15

### Refinement

**Table:**

Refinement on *F*^2^	Primary atom site location: structure-invariant direct methods
Least-squares matrix: full	Secondary atom site location: difference Fourier map
*R*[*F*^2^ > 2σ(*F*^2^)] = 0.042	Hydrogen site location: inferred from neighbouring sites
*wR*(*F*^2^) = 0.088	H-atom parameters constrained
*S* = 1.08	*w* = 1/[σ^2^(*F*_o_^2^) + (0.0261*P*)^2^] where *P* = (*F*_o_^2^ + 2*F*_c_^2^)/3
4156 reflections	(Δ/σ)_max_ < 0.001
289 parameters	Δρ_max_ = 0.48 e Å^−^^3^
0 restraints	Δρ_min_ = −0.60 e Å^−^^3^

### Special details

**Table:**

Geometry. All esds (except the esd in the dihedral angle between two l.s. planes) are estimated using the full covariance matrix. The cell esds are taken into account individually in the estimation of esds in distances, angles and torsion angles; correlations between esds in cell parameters are only used when they are defined by crystal symmetry. An approximate (isotropic) treatment of cell esds is used for estimating esds involving l.s. planes.
Refinement. Refinement of F^2^ against ALL reflections. The weighted R-factor wR and goodness of fit S are based on F^2^, conventional R-factors R are based on F, with F set to zero for negative F^2^. The threshold expression of F^2^ > 2sigma(F^2^) is used only for calculating R-factors(gt) etc. and is not relevant to the choice of reflections for refinement. R-factors based on F^2^ are statistically about twice as large as those based on F, and R- factors based on ALL data will be even larger.

### Fractional atomic coordinates and isotropic or equivalent isotropic displacement parameters (Å^2^)

**Table:**

	*x*	*y*	*z*	*U*_iso_*/*U*_eq_	
O2	0.3003 (3)	0.3205 (3)	0.2453 (2)	0.0369 (7)	
O1	0.8547 (3)	0.5077 (3)	−0.1194 (2)	0.0400 (7)	
N1	1.0759 (4)	0.7489 (3)	−0.1818 (3)	0.0430 (9)	
H27	0.9901	0.7554	−0.1925	0.052*	
C26	0.9626 (4)	0.6411 (4)	−0.0431 (3)	0.0348 (9)	
H26A	0.9904	0.6260	0.0242	0.042*	
H26B	0.9148	0.7041	−0.0297	0.042*	
C25	1.1071 (4)	0.7051 (4)	−0.0904 (3)	0.0374 (10)	
H25A	1.1891	0.7867	−0.0361	0.045*	
H25B	1.1456	0.6359	−0.1118	0.045*	
C23	0.3021 (5)	0.4531 (4)	0.4153 (3)	0.0400 (10)	
H23A	0.3559	0.5439	0.4701	0.048*	
H23B	0.1963	0.4370	0.3904	0.048*	
N2	0.2945 (4)	0.3417 (3)	0.4602 (3)	0.0433 (9)	
H28	0.3363	0.2896	0.4321	0.052*	
C22	0.2227 (5)	0.3152 (4)	0.5466 (3)	0.0412 (10)	
C21	0.1686 (5)	0.4035 (4)	0.6046 (3)	0.0459 (11)	
H21	0.1779	0.4828	0.5844	0.055*	
C17	0.1018 (5)	0.3753 (5)	0.6910 (4)	0.0587 (13)	
H17	0.0663	0.4359	0.7287	0.070*	
C20	0.2069 (6)	0.1977 (5)	0.5794 (4)	0.0620 (14)	
H20	0.2415	0.1362	0.5419	0.074*	
C18	0.0861 (6)	0.2601 (6)	0.7232 (4)	0.0731 (16)	
H18	0.0398	0.2415	0.7818	0.088*	
C19	0.1402 (7)	0.1723 (6)	0.6672 (5)	0.0773 (16)	
H19	0.1316	0.0942	0.6889	0.093*	
Br1	0.11582 (5)	0.01770 (4)	0.13229 (4)	0.05174 (16)	
Br2	0.69190 (6)	0.23673 (5)	−0.29235 (4)	0.06357 (18)	
C16	0.6291 (5)	0.2847 (4)	−0.1613 (3)	0.0361 (10)	
C12	0.2787 (4)	0.1563 (4)	0.0850 (3)	0.0361 (10)	
C14	0.4497 (4)	0.2208 (4)	−0.0404 (3)	0.0335 (9)	
C15	0.4972 (5)	0.1871 (4)	−0.1373 (3)	0.0393 (10)	
H15	0.4386	0.0976	−0.1859	0.047*	
C8	0.6741 (4)	0.4570 (4)	0.0051 (3)	0.0316 (9)	
H8	0.7325	0.5478	0.0521	0.038*	
C11	0.3604 (4)	0.2967 (4)	0.1557 (3)	0.0306 (9)	
C9	0.5388 (4)	0.3599 (4)	0.0326 (3)	0.0309 (9)	
C13	0.3193 (5)	0.1211 (4)	−0.0093 (3)	0.0389 (10)	
H13	0.2607	0.0296	−0.0551	0.047*	
C10	0.4885 (4)	0.3939 (4)	0.1290 (3)	0.0333 (10)	
H10	0.5441	0.4852	0.1756	0.040*	
C4	1.1763 (5)	0.7809 (4)	−0.2529 (3)	0.0355 (10)	
C24	0.3877 (5)	0.4567 (4)	0.3245 (3)	0.0412 (10)	
H24A	0.3936	0.5321	0.2946	0.049*	
H24B	0.4939	0.4732	0.3485	0.049*	
C7	0.7221 (5)	0.4225 (4)	−0.0882 (3)	0.0356 (10)	
C5	1.3276 (5)	0.7942 (4)	−0.2341 (3)	0.0393 (10)	
H5	1.3635	0.7809	−0.1716	0.047*	
C3	1.1295 (5)	0.8053 (4)	−0.3463 (3)	0.0464 (11)	
H3	1.0304	0.8004	−0.3600	0.056*	
C2	1.2272 (6)	0.8364 (5)	−0.4183 (4)	0.0554 (13)	
H2	1.1926	0.8507	−0.4808	0.067*	
C6	1.4243 (5)	0.8267 (4)	−0.3071 (3)	0.0454 (11)	
H6	1.5249	0.8352	−0.2927	0.055*	
C1	1.3768 (5)	0.8471 (4)	−0.4005 (4)	0.0533 (12)	
H1	1.4424	0.8673	−0.4500	0.064*	

### Atomic displacement parameters (Å^2^)

**Table:**

	*U*^11^	*U*^22^	*U*^33^	*U*^12^	*U*^13^	*U*^23^
O2	0.0296 (14)	0.0370 (15)	0.0298 (15)	0.0063 (12)	0.0047 (12)	0.0028 (12)
O1	0.0417 (16)	0.0303 (15)	0.0368 (16)	0.0061 (13)	0.0161 (13)	0.0082 (12)
N1	0.035 (2)	0.056 (2)	0.049 (2)	0.0236 (17)	0.0143 (18)	0.0286 (18)
C26	0.035 (2)	0.032 (2)	0.034 (2)	0.0107 (18)	0.0110 (19)	0.0106 (18)
C25	0.038 (2)	0.033 (2)	0.041 (2)	0.0131 (18)	0.010 (2)	0.0148 (19)
C23	0.042 (2)	0.038 (2)	0.033 (2)	0.014 (2)	0.011 (2)	0.0028 (19)
N2	0.048 (2)	0.049 (2)	0.039 (2)	0.0266 (18)	0.0191 (18)	0.0123 (17)
C22	0.034 (2)	0.044 (3)	0.032 (2)	0.009 (2)	0.004 (2)	0.006 (2)
C21	0.046 (3)	0.041 (3)	0.038 (3)	0.012 (2)	0.010 (2)	0.006 (2)
C17	0.060 (3)	0.052 (3)	0.041 (3)	0.012 (2)	0.016 (2)	−0.001 (2)
C20	0.069 (3)	0.057 (3)	0.067 (4)	0.029 (3)	0.024 (3)	0.025 (3)
C18	0.081 (4)	0.071 (4)	0.048 (3)	0.016 (3)	0.028 (3)	0.018 (3)
C19	0.100 (5)	0.068 (4)	0.079 (4)	0.039 (3)	0.040 (4)	0.043 (3)
Br1	0.0463 (3)	0.0404 (3)	0.0531 (3)	0.0058 (2)	0.0203 (2)	0.0115 (2)
Br2	0.0729 (4)	0.0480 (3)	0.0541 (3)	0.0154 (3)	0.0345 (3)	0.0052 (2)
C16	0.046 (2)	0.032 (2)	0.027 (2)	0.0146 (19)	0.0151 (19)	0.0076 (17)
C12	0.027 (2)	0.033 (2)	0.041 (3)	0.0069 (17)	0.0077 (19)	0.0128 (19)
C14	0.035 (2)	0.029 (2)	0.031 (2)	0.0097 (18)	0.0061 (19)	0.0077 (17)
C15	0.042 (2)	0.030 (2)	0.038 (3)	0.0110 (19)	0.009 (2)	0.0060 (19)
C8	0.031 (2)	0.028 (2)	0.034 (2)	0.0127 (18)	0.0050 (18)	0.0075 (17)
C11	0.025 (2)	0.036 (2)	0.027 (2)	0.0116 (18)	0.0038 (18)	0.0083 (18)
C9	0.032 (2)	0.031 (2)	0.031 (2)	0.0150 (18)	0.0061 (18)	0.0112 (17)
C13	0.035 (2)	0.031 (2)	0.038 (3)	0.0066 (18)	0.006 (2)	0.0037 (19)
C10	0.033 (2)	0.030 (2)	0.032 (2)	0.0121 (18)	0.0004 (19)	0.0052 (17)
C4	0.039 (2)	0.026 (2)	0.034 (2)	0.0087 (18)	0.010 (2)	0.0063 (17)
C24	0.040 (2)	0.037 (2)	0.034 (2)	0.0096 (19)	0.006 (2)	0.0058 (19)
C7	0.038 (2)	0.027 (2)	0.039 (3)	0.0109 (19)	0.008 (2)	0.0121 (18)
C5	0.035 (2)	0.040 (2)	0.044 (3)	0.0163 (19)	0.009 (2)	0.016 (2)
C3	0.035 (2)	0.050 (3)	0.043 (3)	0.008 (2)	0.002 (2)	0.020 (2)
C2	0.055 (3)	0.063 (3)	0.038 (3)	0.015 (2)	0.005 (2)	0.021 (2)
C6	0.041 (3)	0.041 (2)	0.052 (3)	0.016 (2)	0.017 (2)	0.014 (2)
C1	0.054 (3)	0.052 (3)	0.044 (3)	0.013 (2)	0.019 (2)	0.014 (2)

### Geometric parameters (Å, º)

**Table:**

O2—C11	1.360 (4)	Br2—C16	1.889 (4)
O2—C24	1.442 (4)	C16—C15	1.368 (5)
O1—C7	1.367 (5)	C16—C7	1.426 (5)
O1—C26	1.440 (4)	C12—C13	1.339 (5)
N1—C4	1.375 (5)	C12—C11	1.424 (5)
N1—C25	1.442 (4)	C14—C15	1.399 (5)
N1—H27	0.8600	C14—C13	1.416 (5)
C26—C25	1.505 (5)	C14—C9	1.430 (5)
C26—H26A	0.9700	C15—H15	0.9300
C26—H26B	0.9700	C8—C7	1.362 (5)
C25—H25A	0.9700	C8—C9	1.408 (5)
C25—H25B	0.9700	C8—H8	0.9300
C23—N2	1.442 (5)	C11—C10	1.362 (5)
C23—C24	1.486 (6)	C9—C10	1.407 (5)
C23—H23A	0.9700	C13—H13	0.9300
C23—H23B	0.9700	C10—H10	0.9300
N2—C22	1.384 (5)	C4—C3	1.392 (5)
N2—H28	0.8600	C4—C5	1.392 (5)
C22—C21	1.386 (6)	C24—H24A	0.9700
C22—C20	1.392 (6)	C24—H24B	0.9700
C21—C17	1.365 (6)	C5—C6	1.373 (6)
C21—H21	0.9300	C5—H5	0.9300
C17—C18	1.365 (7)	C3—C2	1.368 (6)
C17—H17	0.9300	C3—H3	0.9300
C20—C19	1.375 (7)	C2—C1	1.386 (6)
C20—H20	0.9300	C2—H2	0.9300
C18—C19	1.371 (7)	C6—C1	1.371 (6)
C18—H18	0.9300	C6—H6	0.9300
C19—H19	0.9300	C1—H1	0.9300
Br1—C12	1.902 (4)		
			
C11—O2—C24	117.5 (3)	C11—C12—Br1	117.5 (3)
C7—O1—C26	117.5 (3)	C15—C14—C13	122.7 (3)
C4—N1—C25	123.3 (3)	C15—C14—C9	119.1 (4)
C4—N1—H27	118.3	C13—C14—C9	118.1 (4)
C25—N1—H27	118.3	C16—C15—C14	120.6 (3)
O1—C26—C25	107.3 (3)	C16—C15—H15	119.7
O1—C26—H26A	110.3	C14—C15—H15	119.7
C25—C26—H26A	110.3	C7—C8—C9	122.2 (3)
O1—C26—H26B	110.3	C7—C8—H8	118.9
C25—C26—H26B	110.3	C9—C8—H8	118.9
H26A—C26—H26B	108.5	O2—C11—C10	126.0 (3)
N1—C25—C26	112.2 (3)	O2—C11—C12	115.4 (3)
N1—C25—H25A	109.2	C10—C11—C12	118.6 (4)
C26—C25—H25A	109.2	C10—C9—C8	122.7 (3)
N1—C25—H25B	109.2	C10—C9—C14	118.8 (4)
C26—C25—H25B	109.2	C8—C9—C14	118.4 (4)
H25A—C25—H25B	107.9	C12—C13—C14	121.1 (4)
N2—C23—C24	110.3 (3)	C12—C13—H13	119.4
N2—C23—H23A	109.6	C14—C13—H13	119.4
C24—C23—H23A	109.6	C11—C10—C9	121.7 (3)
N2—C23—H23B	109.6	C11—C10—H10	119.1
C24—C23—H23B	109.6	C9—C10—H10	119.1
H23A—C23—H23B	108.1	N1—C4—C3	119.9 (4)
C22—N2—C23	122.7 (3)	N1—C4—C5	122.5 (4)
C22—N2—H28	118.7	C3—C4—C5	117.5 (4)
C23—N2—H28	118.7	O2—C24—C23	106.8 (3)
N2—C22—C21	122.6 (4)	O2—C24—H24A	110.4
N2—C22—C20	119.5 (4)	C23—C24—H24A	110.4
C21—C22—C20	117.9 (4)	O2—C24—H24B	110.4
C17—C21—C22	120.7 (4)	C23—C24—H24B	110.4
C17—C21—H21	119.6	H24A—C24—H24B	108.6
C22—C21—H21	119.6	C8—C7—O1	126.1 (3)
C18—C17—C21	121.5 (5)	C8—C7—C16	118.6 (4)
C18—C17—H17	119.3	O1—C7—C16	115.3 (4)
C21—C17—H17	119.3	C6—C5—C4	120.6 (4)
C19—C20—C22	120.2 (5)	C6—C5—H5	119.7
C19—C20—H20	119.9	C4—C5—H5	119.7
C22—C20—H20	119.9	C2—C3—C4	120.8 (4)
C17—C18—C19	118.5 (5)	C2—C3—H3	119.6
C17—C18—H18	120.7	C4—C3—H3	119.6
C19—C18—H18	120.7	C3—C2—C1	121.5 (4)
C18—C19—C20	121.2 (5)	C3—C2—H2	119.2
C18—C19—H19	119.4	C1—C2—H2	119.2
C20—C19—H19	119.4	C1—C6—C5	121.9 (4)
C15—C16—C7	121.0 (4)	C1—C6—H6	119.1
C15—C16—Br2	120.0 (3)	C5—C6—H6	119.1
C7—C16—Br2	119.0 (3)	C6—C1—C2	117.5 (4)
C13—C12—C11	121.4 (4)	C6—C1—H1	121.2
C13—C12—Br1	121.0 (3)	C2—C1—H1	121.2
			
C7—O1—C26—C25	172.5 (3)	C13—C14—C9—C8	176.4 (3)
C4—N1—C25—C26	−165.2 (3)	C11—C12—C13—C14	2.8 (6)
O1—C26—C25—N1	68.6 (4)	Br1—C12—C13—C14	−174.2 (3)
C24—C23—N2—C22	179.3 (3)	C15—C14—C13—C12	178.8 (4)
C23—N2—C22—C21	−7.7 (6)	C9—C14—C13—C12	1.0 (6)
C23—N2—C22—C20	174.1 (4)	O2—C11—C10—C9	−178.1 (3)
N2—C22—C21—C17	−178.4 (4)	C12—C11—C10—C9	1.7 (5)
C20—C22—C21—C17	−0.1 (6)	C8—C9—C10—C11	−177.7 (3)
C22—C21—C17—C18	0.1 (7)	C14—C9—C10—C11	2.1 (5)
N2—C22—C20—C19	178.0 (4)	C25—N1—C4—C3	172.0 (4)
C21—C22—C20—C19	−0.3 (7)	C25—N1—C4—C5	−9.8 (6)
C21—C17—C18—C19	0.5 (8)	C11—O2—C24—C23	−177.4 (3)
C17—C18—C19—C20	−0.9 (8)	N2—C23—C24—O2	60.5 (4)
C22—C20—C19—C18	0.9 (8)	C9—C8—C7—O1	−177.2 (3)
C7—C16—C15—C14	0.5 (6)	C9—C8—C7—C16	2.0 (5)
Br2—C16—C15—C14	179.7 (3)	C26—O1—C7—C8	6.6 (5)
C13—C14—C15—C16	−176.4 (4)	C26—O1—C7—C16	−172.6 (3)
C9—C14—C15—C16	1.4 (6)	C15—C16—C7—C8	−2.1 (6)
C24—O2—C11—C10	−6.2 (5)	Br2—C16—C7—C8	178.6 (3)
C24—O2—C11—C12	174.0 (3)	C15—C16—C7—O1	177.1 (3)
C13—C12—C11—O2	175.6 (3)	Br2—C16—C7—O1	−2.1 (5)
Br1—C12—C11—O2	−7.3 (4)	N1—C4—C5—C6	−179.8 (4)
C13—C12—C11—C10	−4.2 (6)	C3—C4—C5—C6	−1.6 (6)
Br1—C12—C11—C10	172.9 (3)	N1—C4—C3—C2	−179.6 (4)
C7—C8—C9—C10	179.6 (3)	C5—C4—C3—C2	2.1 (6)
C7—C8—C9—C14	−0.2 (5)	C4—C3—C2—C1	−1.1 (7)
C15—C14—C9—C10	178.7 (3)	C4—C5—C6—C1	−0.1 (6)
C13—C14—C9—C10	−3.4 (5)	C5—C6—C1—C2	1.2 (6)
C15—C14—C9—C8	−1.5 (5)	C3—C2—C1—C6	−0.6 (7)

### Hydrogen-bond geometry (Å, º)

Cg1 and Cg2 are the centroids of the C1–C6 and C9–C14 benzene rings, respectively.

**Table:**

*D*—H···*A*	*D*—H	H···*A*	*D*···*A*	*D*—H···*A*
N1—H27···Br1^i^	0.86	3.03	3.705 (4)	137
N1—H27···O2^i^	0.86	2.53	3.349 (4)	160
C5—H5···*Cg*2^ii^	0.93	2.81	3.626 (5)	147
C15—H15···*Cg*1^iii^	0.93	2.86	3.782 (4)	175

Symmetry codes: (i) −*x*+1, −*y*+1, −*z*; (ii) −*x*+2, −*y*+1, −*z*; (iii) *x*−1, *y*−1, *z*.

## References

[bb1] Bruker (2007). *SMART* and *SAINT* Bruker AXS Inc., Madison, Wisconsin, USA.

[bb2] Chandan, S., Ved, P. V. & Kumar, P. S. (2008). PCT Int. Appl. 2008099415.

[bb3] Chandan, S., Ved, P. V., Niraj, K. N., Ajit, S. S., Mohammad, H. & Sunil, K. P. (2008). *J. Med. Chem.* **51**, 1313–1315.

[bb4] Liou, G. S., Lin, P. H., Yen, H. J., Yu, Y. Y. & Chen, W. C. (2011). *J. Poly. Sci. Part A*, **48**, 1433–1440.

[bb5] Qin, W. W., Zhnag, Y. L., Liu, W. S. & Tan, M. Y. (2003). *Spectrochim. Acta Part A*, **59**, 3085–3092. 10.1016/s1386-1425(03)00112-4 14583283

[bb6] Sheldrick, G. M. (1996). *SADABS* University of Göttingen, Germany.

[bb7] Sheldrick, G. M. (2008). *Acta Cryst.* A**64**, 112–122. 10.1107/S0108767307043930 18156677

[bb8] Tan, G. Z., Xu, J. Z. & Jiao, T. Q. (1986). *Chin. J. Org. Chem* **2**, 143–145.

[bb9] Zhang, Y. L., Qin, W. W., Liu, W. S., Tan, M. Y. & Tang, N. (2002). *Spectrochim. Acta Part A*, **58**, 2153–2157. 10.1016/s1386-1425(01)00680-1 12212740

